# Lead Screening for CXCR4 of the Human HIV Infection Receptor Inhibited by Traditional Chinese Medicine

**DOI:** 10.1155/2014/809816

**Published:** 2014-06-05

**Authors:** Tzu-Chieh Hung, Wen-Yuan Lee, Kuen-Bao Chen, Calvin Yu-Chian Chen

**Affiliations:** ^1^Department of Biomedical Informatics, Asia University, Taichung 41354, Taiwan; ^2^School of Medicine, College of Medicine, China Medical University, Taichung 40402, Taiwan; ^3^Department of Neurosurgery, China Medical University Hospital, No. 2, Yude Road, North District, Taichung City 40447, Taiwan; ^4^Department of Anesthesiology, China Medical University Hospital, Taichung 40447, Taiwan; ^5^Research Center for Chinese Medicine & Acupuncture, China Medical University, Taichung 40402, Taiwan

## Abstract

The acquired immunodeficiency syndrome (AIDS) is a serious worldwide disease caused by the human immunodeficiency virus (HIV) infection. Recent research has pointed out that the G protein-coupled chemokine receptor CXCR4 and the coreceptor C-C chemokine receptor type 5 (CCR5) are important targets for HIV infection. The traditional Chinese medicine (TCM) database has been screened for candidate compounds by simulating molecular docking and molecular dynamics against HIV. Saussureamine C, 5-hydroxy-L-tryptophan, and diiodotyrosine are selected based on the highest docking score. The molecular dynamics is helpful in the analysis and detection of protein-ligand interactions. According to the analysis of docking poses, hydrophobic interactions, hydrogen bond variations, and the comparison of the effect on CXCR4 and CCR5, these results indicate Saussureamine C may have better effect on these two receptors. But for some considerations, diiodotyrosine could make the largest variation and may have some efficacy contrary to expectations.

## 1. Introduction

Recently, an important topic of the acquired immunodeficiency syndrome (AIDS) had been published in 2013. The G protein-coupled chemokine receptor CXCR4 and the coreceptor C-C chemokine receptor type 5 (CCR5) are important targets for HIV infection [1].

The human immunodeficiency virus (HIV) is a retrovirus which causes the AIDS [2–5]. During the course of this disease, the human immune system becomes compromised, and considerably weaker. The HIV virus is transmitted through a population rapidly by unprotected sexual intercourse [6, 7], contaminated medical equipment [8, 9], vertical infection [10, 11], and bodily fluids.

There were 35.3 million people living with HIV in 2012 and 2.1 million of these patients were adolescents (recorded by WHO). There are still no defined vaccines or drugs available to kill all HIV viruses in patients and then the highly active antiretroviral therapy (HAART) is the standard of care for patients with advanced infection in current treatment [12]. HARRT uses a complex of transcription inhibitors to slow down transcription and then decrease the patient's total burden of HIV, but this treatment is too expensive and medical costs become a social liability.

Chemokine receptors are critical regulators of cell migration in the context of immune surveillance, inflammation, and development. The one of 19 known human chemokine receptors, the G protein-coupled chemokine receptor CXCR4, is specifically implicated in cancer metastasis and HIV-1 infection [13]. The CCR5 is a receptor for the T-cells that play a central role in cell-mediated immunity against viruses and pathogens. CXCR4 and CCR5 have been defined as coreceptors for the HIV antigen gp120 and then HIV can infect the cell by targeting these receptors [14, 15]. Thus, preventing HIV from targeting the receptor could prevent the virus infection [16, 17]. Based on these observations, the drug IT1t is a CCR5 receptor antagonist, thereby blocking the HIV protein from associating with the receptor.

Computer-aided drug design (CADD) is an* in silico* simulation technique to screen for novel compounds by their structure and bioactivity from database. The difference from traditional drug design is that CADD has the advantages of both greater speed and lower cost for drug development. The structure-based drug design and ligand-based drug design are two major application areas of CADD. We used CADD to investigate based on structure-based drug design and molecular dynamics [18–21].

Recently, more attention has been given to personalized medicine and biomedicine [22, 23]. By this knowledge, people could discover the association from the mutation [24, 25], pathway [26, 27], the cause for special disease [28–30], and even the case from clinical diagnosis [31] with disease. Traditional Chinese medicine (TCM) is an identified personalized medicine and this clinical diagnosis has an important role in Asia, especially in China, Taiwan, Korea, and Japan. In 2011, the TCM Database@Taiwan (http://tcm.cmu.edu.tw/) [32] which is the largest traditional Chinese medicine database in the world was established. In this TCM database, both of 2D and 3D chemical structures, bioactivity, and molecular information for over 61,000 compounds of traditional Chinese medicinal herbs could be generated. Until today, there has been successful novel drug discovery from the TCM Database@Taiwan, such as cancer treatment [33–36], stroke prevention [37], EGFR inhibition [38], inflammation inhibition [39], pain relief [19], and antivirals [40–43]. Since the application system of the website [44] and the cloud computing platform [45], the TCM Database@Taiwan could be rigorous and valuable for TCM application and drug design.

In this research, we screen a possible compound against HIV from the TCM Database@Taiwan based on molecular docking. Finally, we use molecular dynamics (MD) simulation to investigate the protein-ligand interactions that may contribute to evaluate the effect of human HIV receptor inhibition.

## 2. Materials and Methods

### 2.1. Data Set

A total of 61,000 TCM compounds were downloaded from the TCM database (http://tcm.cmu.edu.tw/). The CXCR4 (PDB ID: 3ODU) crystal structure was generated from RCSB Protein Data Bank (PDB). Based on the literature, IT1t was defined as a control [13]. Then, the Accelrys Discovery Studio 2.5 (DS 2.5) was used for the molecular docking selection.

### 2.2. Disorder Protein Detection

Because the disorder protein plays an important role in drug design, we take the crystal structure to predict the disorder region by the Database of Protein Disorder (DisProt: http://www.disprot.org/). Based on the prediction, we can decide the character of the docking site and assess the efficacy of the drug [46, 47].

After a comparison of the disorder regions and the defined docking sites, we could evaluate drug efficacy from the protein-ligand interaction.

### 2.3. Molecular Docking

The docking platform dock IT1t and TCM compounds to CXCR4 in the force field of CHARMm [48] by using LigandFit [49], which is a receptor-rigid docking algorithm in Discovery Studio 2.5 (DS 2.5). The docking site of CXCR4 was defined by the research around Trp94, Asp97, Trp102, Val112, His113, Tyr116, Arg183, Cys186, Asp187, Arg188, and Glu288 [1, 13]. The complexes of candidate compounds with CXCR4 were selected for hydrophobic interactions by Ligplus [50, 51].

### 2.4. Molecular Dynamics Simulation

These selected ligands must take preparation by using SwissParam (http://swissparam.ch/) [52] before MD simulation based on the reference force field [53] of GROMACS 4.5.5 [54]. The CXCR4 combines with ligands as the complex set into the full buffer (or solution) simulation box. This cubic box was set with a minimum distance of 1.2 Å from the complex and in this simulation box was solvated with the TIP3P water model to regulate the sodium and chloride ion to neutralize complex charges. The minimization applies the steepest descent method for 5,000 steps in the beginning. Then the last structure was transferred to MD simulation. The equilibration was based on the Berendsen weak thermal coupling method under the 100 ps constant temperature (PER ensemble). The Particle-Mesh Ewald (PME) [55] method was used to calculate the electrostatic interactions in 2 fs per time and the numbers of steps were 5,000,000 times and then accomplished 10 ns simulation time of MD. Gromacs 4.5.5 also has protocol to analyze MD trajectories, RMSD, and energy variations.

## 3. Results and Discussion

### 3.1. The Detection of Disorder Protein

**Figure 1:**
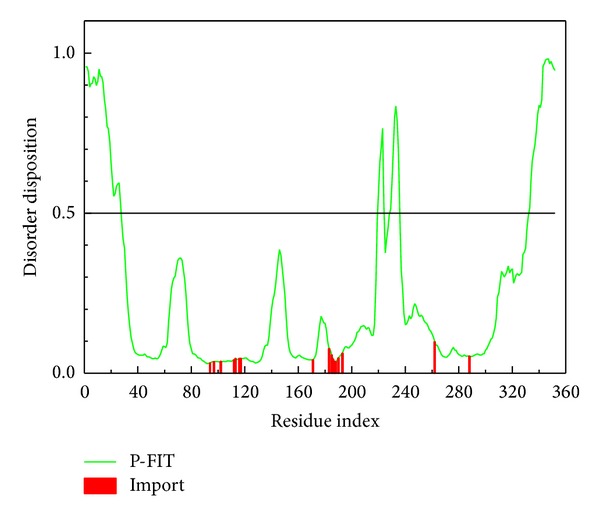
The disorder and binding site detection. The green curve in the figure is the disorder disposition of each amino acid and the red lines are the residues of the important amino acids.

The disorder protein is defined as unfolding protein. For this character, while the drug is docking to the disorder region, the complex will stabilize with difficulty. There are some references [46, 47] that indicate that the disorder region is not any defined domain; therefore, therefore the drug targeting the disorder region may have lower side effects than a drug interacting with the widespread domain. Thus, the disorder region can be defined as a hard work for drug design. The disorder regions of CXCR4 are defined as having a disposition of over than 0.5 (Figure 1). This result indicates that the important amino acids are not disorder regions; thus, the complex selected based on docking could have an influence on CXCR4.

### 3.2. Molecular Docking

**Figure 2:**
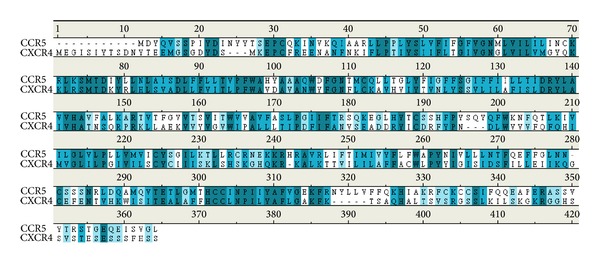
The sequence analysis between two HIV influence receptors. The deep blue of sequence means these two regions are the same, low blue means similar, and white means different.

After molecular docking and ranking by docking score, the top three TCM compounds are defined as candidate compounds which are Saussureamine C, 5-hydroxy-L-tryptophan, and diiodotyrosine derived from the TCM herbs* Saussurea lappa *Clarke,* Mucuna pruriens *(L) D., and* Ox Thyroid of Bos taurus domesticus Gmelin* (or* Bubalus bubalis* L.), respectively (Table 1). The top ranking compound, Saussureamine C is know for anti-ulcer treatment [56] and the herb* Saussurea lappa *Clarke can inhibit breast cancer migration [57], treat cardiovascular disease [58, 59], be used against hepatotoxic activity [60], and inhibit cytotoxic T lymphocytes [61]. The second ranked herb,* Mucuna pruriens*, can prevent Parkinson's disease based on antioxidation [62, 63]. The third ranked compound, diiodotyrosine from the herb* Ox Thyroid of Bos taurus domesticus Gmelin*, has been related to the thyroid [64–66] and pH-sensitive pore-forming [67]. As reported in the literature, most of these compounds can have an effect on immunity, especially on cancer. For the above reference, we suggest that these compounds can have an effect on T-cell receptors, such as CXCR4. For our previous research about the coreceptor CCR5, the top and second compounds are the same. We make the sequence align between CXCR4 and CCR5 (Figure 2). This result indicates that the most important amino acids both of CXCR4 and CCR5 are similar and presents that the docking site and binding domain designed by important amino acids are similar. For this result, the drug has an influence on CXCR4 which may also have an effect on CCR5.

**Figure 3:**
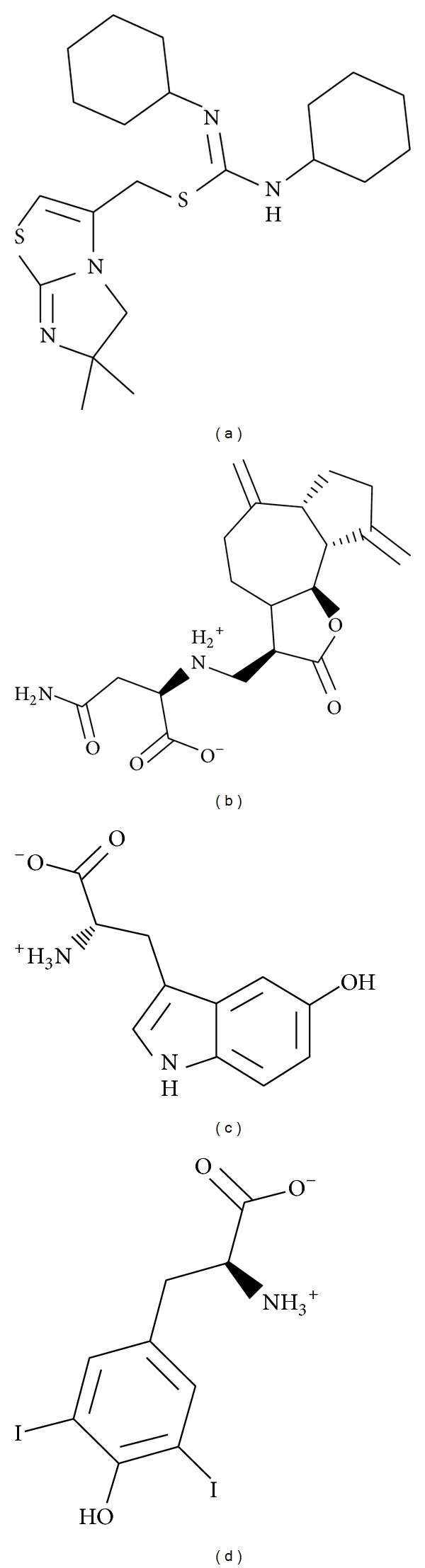
The structure of control and candidate TCM compounds. (a) IT1t, (b) Saussureamine C, (c) 5-hydroxy-L-tryptophan, and (d) diiodotyrosine.

**Figure 4:**
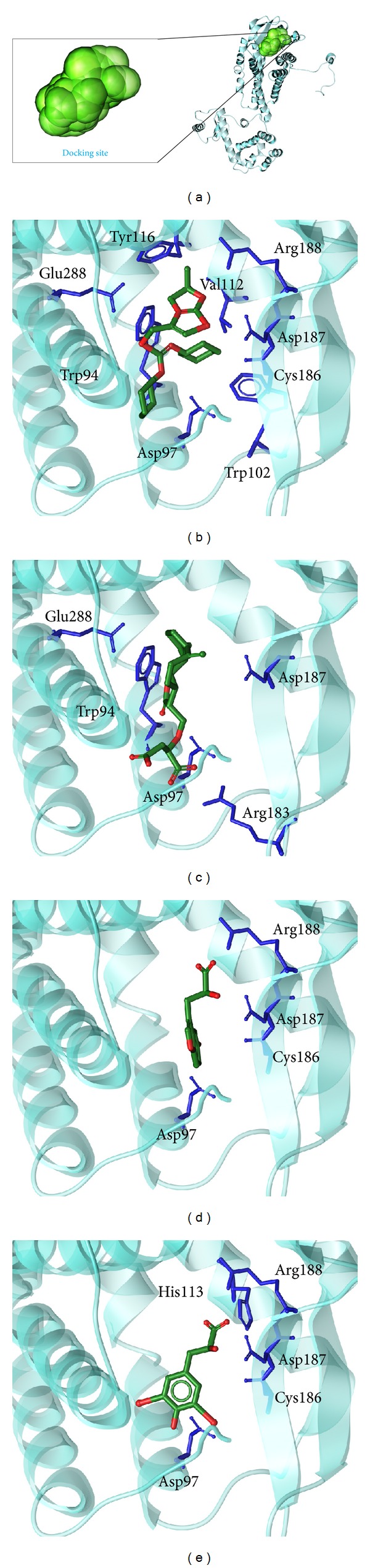
The docking poses of ligands. (a) The crystal structure of CXCR4 and the docking site, (b) IT1t, (c) Saussureamine C, (d) 5-hydroxy-L-tryptophan, and (e) diiodotyrosine.

The structures of the candidate compounds and control were screened from TCM database (Figure 3). Then, the docking poses, the docking site, and the amino acid neighbors by ligands are presented (Figure 4). From this result, we observe that Asp97 and Asp187 are defined as the amino acids that can interact with all the selected ligands; thus, these amino acids may play important roles in target function of CXCR4.

**Figure 5:**
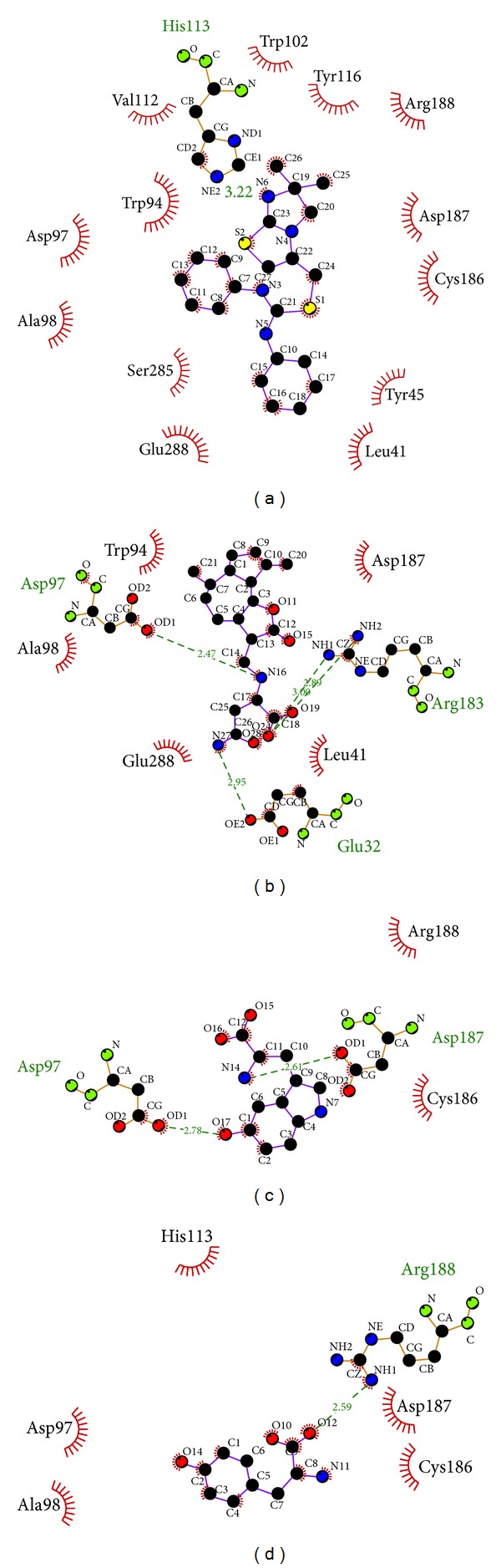
Ligplot illustrates the protein-ligand interactions. (a) IT1t, (b) Saussureamine C, (c) 5-hydroxy-L-tryptophan, and (d) diiodotyrosine. The deep red color of the hydrophobic interactions indicates a high frequency in all ligand interactions.

The hydrophobic interaction can be analyzed by Ligplus (Figure 5). This result shows that the signed deep red amino acids are at a high frequency while ligands target in docking site. Most of these amino acids have been defined as important amino acids in the literature; thus, this hydrophobic interaction analysis is credible to present the selected compounds effect on CXCR4.

### 3.3. Molecular Dynamics Simulation

**Figure 6:**
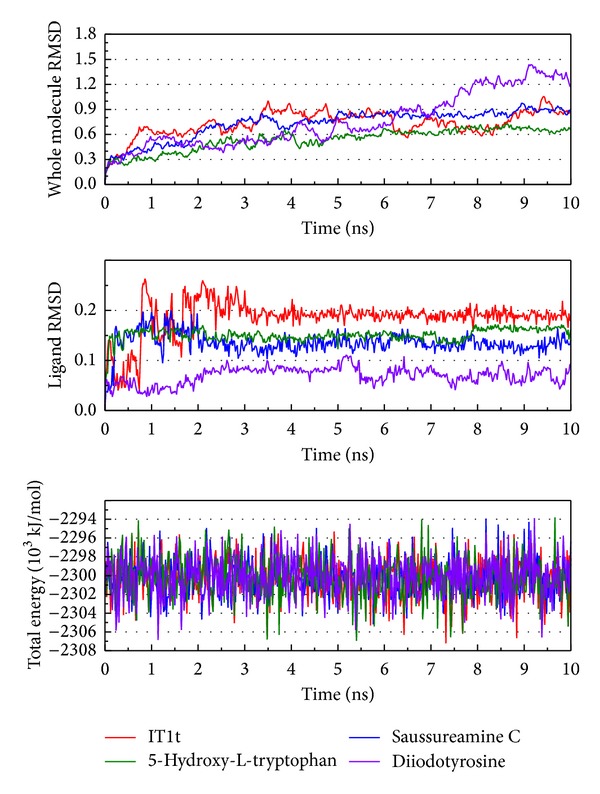
Measures of the MD trajectories. (a) Complex RMSD, (b) ligand RMSD, and (c) the total energy.

The RMSD and total energy of a complex during MD simulation were calculated (Figure 6). The total energy is in the range −2308~−2294∗10^3^ kJ/mol and tends to −2300∗10^3^ kJ/mol. Although the compounds Saussureamine C and 5-hydroxy-L-tryptophan have a high variation in ligand RMSD, the amplitude in both ligand RMSD and protein RMSD is more gentle after MD 8 ns. Thus, we suggest that these two ligands may make the complex balance quickly.

**Figure 7:**
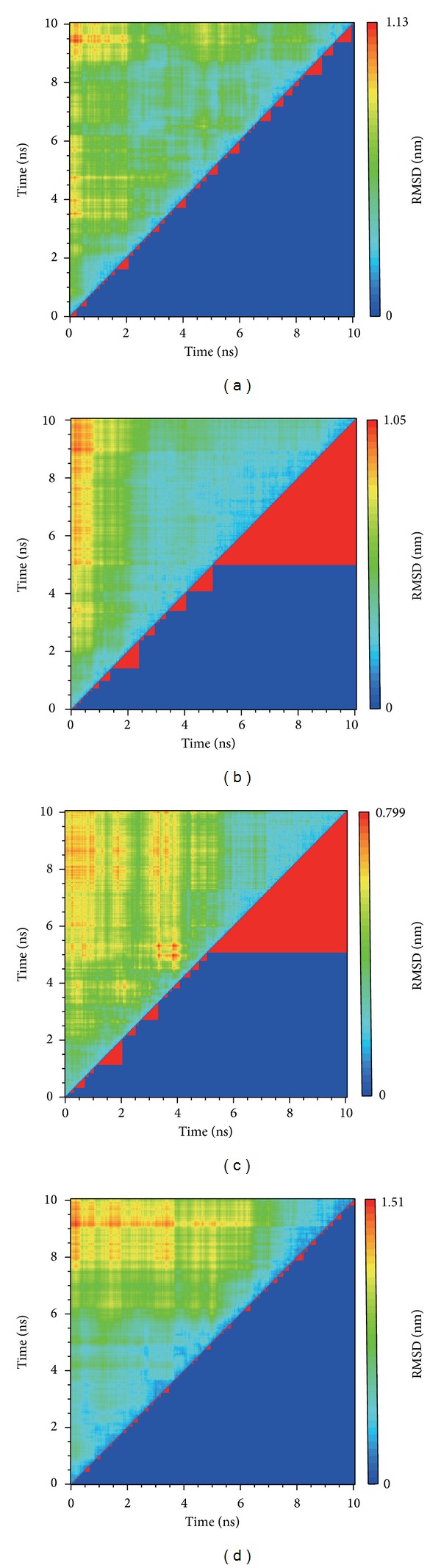
The clustering based on RMSD variation while protein interaction. (a) IT1t, (b) Saussureamine C, (c) 5-Hydroxy-L-tryptophan, (d) Diiodotyrosine.

The clustering based on RMSD variation could be calculated (Figure 7). In this result, the complex with compound Saussureamine C or 5-hydroxy-L-tryptophan and the protein structure position will be similar to the same group after MD 5 ns. This result confirms our suggestion and presents that these two ligands will be fit to CXCR4.

**Figure 8:**
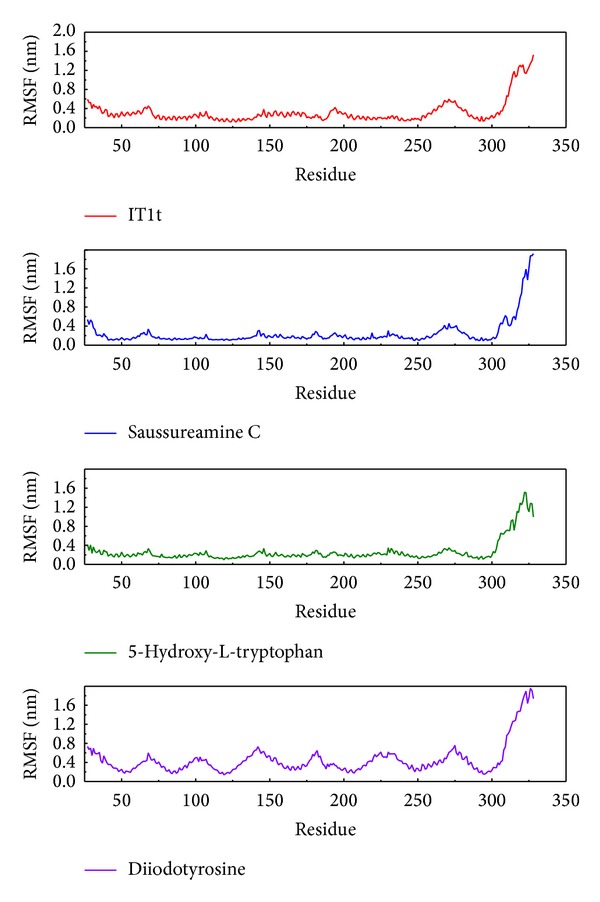
The RMSF of each residue of protein.

The calculations of RMSD in each residue during the whole MD, the root mean square fluctuations (RMSF), show the variation in CXCR4 (Figure 8). In this result, the pick sites of residue in four complexes are similar, and then the pick in residues 50 to 300 could help to define the relation in interaction. The similar pick sites present these compounds effect on the same amino acids and these amino acids may play important function in CXCR4. The largest amplitude of the complex with diiodotyrosine may indicate that this compound will have stronger effect on protein making the complex unstable.

**Figure 9:**
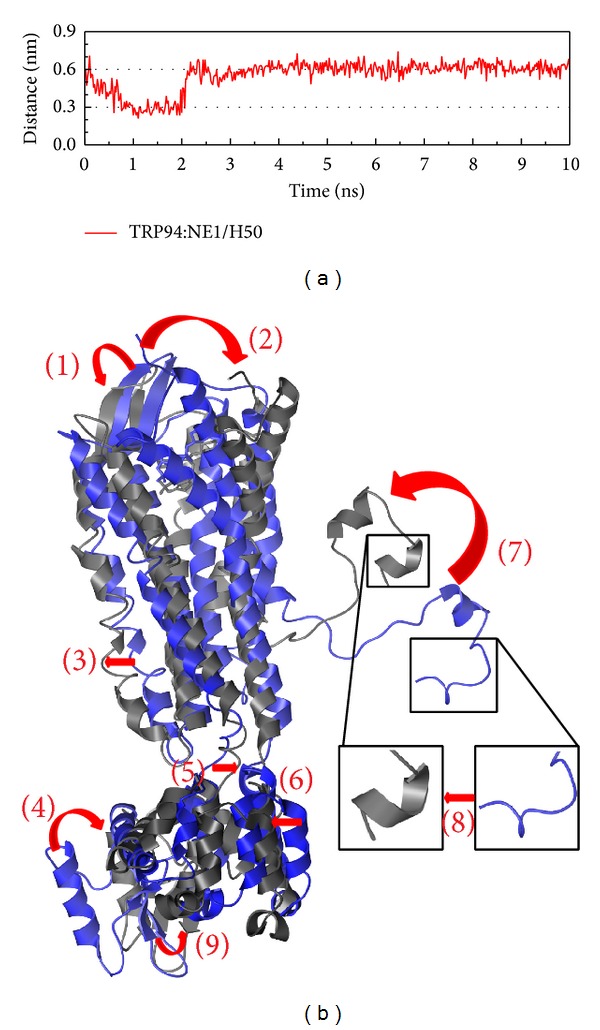
The variation of control and CXCR4 complex in MD simulation. (a) H-bond variation, (b) structure variation. The (1)–(9) red color indicates the difference through MD.

**Figure 10:**
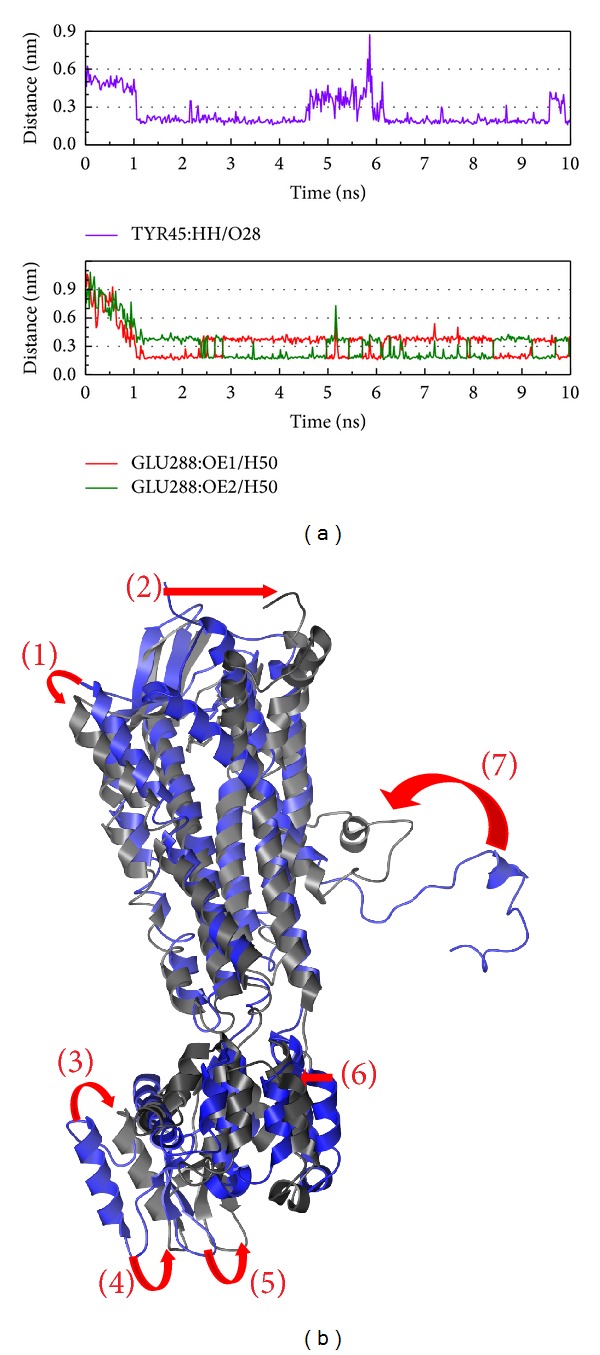
The variation of Saussureamine C and CXCR4 complex in MD simulation. (a) H-bond variation, (b) structure variation. The (1)–(7) red color indicates the difference through MD.

**Figure 11:**
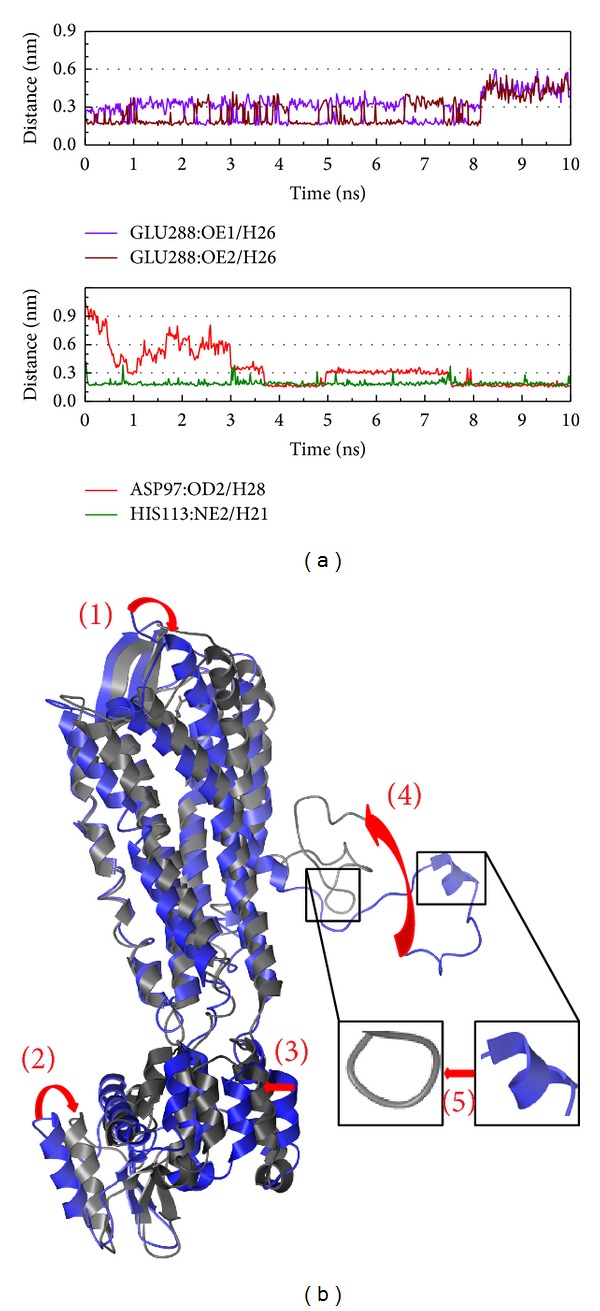
The variation of 5-hydroxy-L-tryptophan and CXCR4 complex in MD simulation. (a) H-bond variation, (b) structure variation. The (1)–(5) red color indicates the difference through MD.

**Figure 12:**
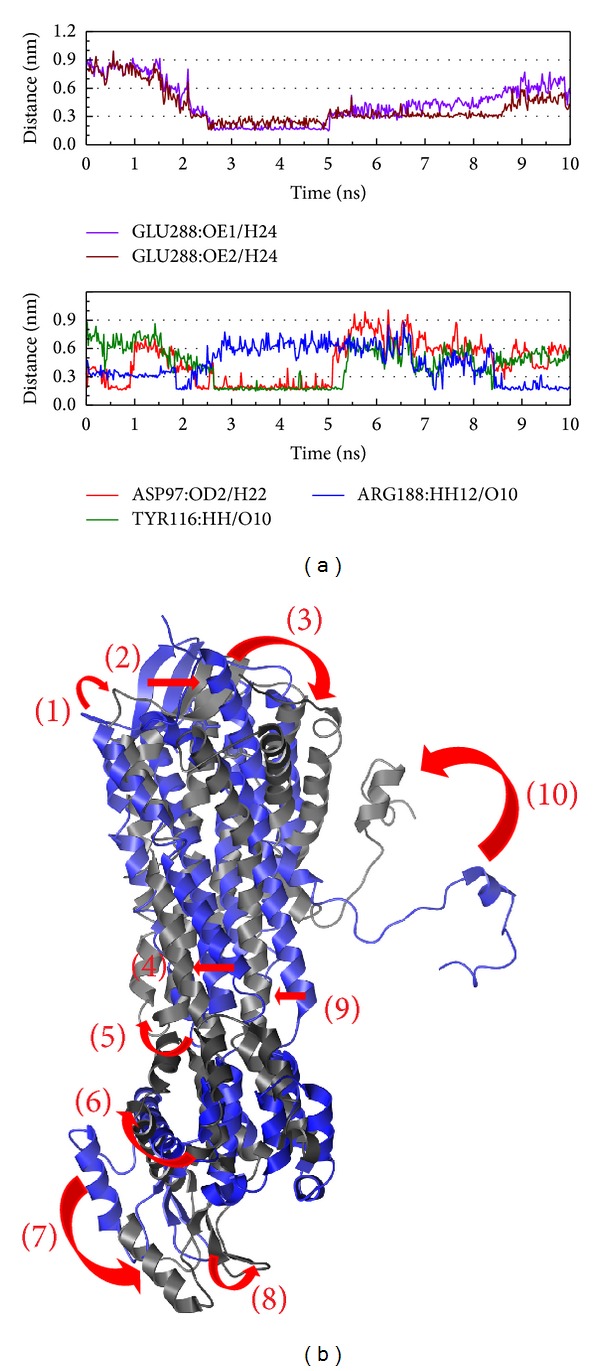
The variation of diiodotyrosine and CXCR4 complex in MD simulation. (a) H-bond variation, (b) structure variation. The (1)–(10) red color indicates the difference through MD.

The H-bond occupancy and structure variation were calculated for the protein-ligand interactions (Figures 9–12). In the complex with control, the H-bond occupancy is less than 10% (Figure 9(a)), but there was still variation in both the position and the composition (Figures 9(b)-8). These variations, we suggest, are due to the complex having more hydrophobic interactions than others might have. This is an important function, while protein-ligand interactions then inhibit the influence of the G protein.

In Figure 10(a), the different atoms of Glu288 interact with ligand after 1000 ps. Besides composition variation, the structure variations of CXCR4 with Saussureamine C are similar to the control (Figure 10(b)). Although the hydrophobic interaction are fewer than the control, the binding site Glu288, for chemokine (defined by Uniprot), will be targeted by Saussureamine C to replace the force of the hydrophobic interaction.

The high H-bond occupancy for CXCR4 with 5-hydroxy-L-tryptophan is not only Glu288 (Figure 11(a)). The Asp97 and His113 had been defined as chemokine binding site by the UniProt; the ligand interacts with these functional sites may cause the structure to loose the helix, which will effect the function of the G protein (Figure 11(b)).

The larger variation in both H-bond and structure for the Diiodotyrosine complex might indicate that this complex does not tend to balance (Figure 12). This situation might indicate that diiodotyrosine is a compound with stronger force and long term interaction for CXCR4. For this possible suggestion, diiodotyrosine might not be bad for the inhibition of CXCR4 but using diiodotyrosine it may have some consideration or supporting measures.

The difference from CCR5 is that the structure variation in CXCR4 is more intense, while the protein-ligand interaction and the ligand are the same. Accordingly, we suggest the ligand inhibits these two receptors which might be intense interaction to change CXCR4 structure and strong target to prevent other influence on CCR5.

**Figure 13:**
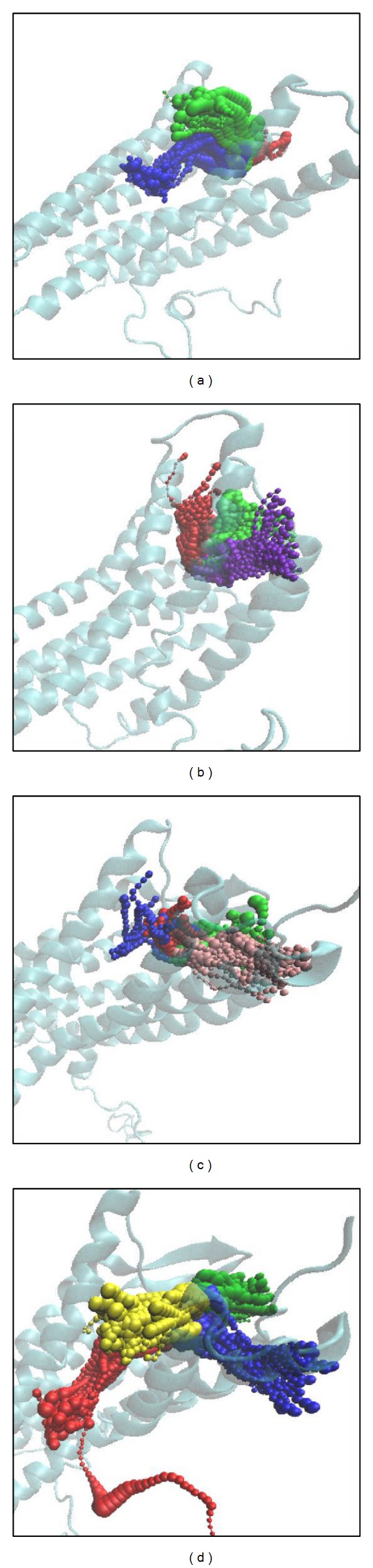
The pathway calculation of CXCR4 complex in MD simulation. (a) IT1t, (b) Saussureamine C, (c) 5-hydroxy-L-tryptophan, and (d) diiodotyrosine.

The pathway definition is according to the calculation of caver 3.0 [68] to determine the interpath in protein while interacting (Figure 13). In this result, we could find most pathways around the docking site, and only diiodotyrosine is different. This phenomenon may be caused by larger structural variations making some pathways hidden to the protein as the virus enters the human cell. Although we could not identify the existence of this hypothesized pathway, we note that Diiodotyrosine is different from the other selected compounds.

## 4. Conclusion

Based on the above discussion, we have seen TCM compounds Saussureamine C, 5-hydroxy-L-tryptophan, and diiodotyrosine can have an effect on CXCR4 against HIV infection. The control interacts with CXCR4 by more hydrophobic interactions but with other compounds on the basis of H-bond, a stronger force in the interaction. The structural variations in CXCR4, with the same compounds, being more intense than in CCR5 might be due to the different modes of protein inhibition. Although Saussureamine C is the best selection from drug design, diiodotyrosine might have some efficacy contrary to expectations from the largest variation.

## Figures and Tables

**Table 1:** Scoring functions of the top three compounds and the inhibitors of CXCR4.

Compounds	Herbs	-PLP1	-PLP2	Dock score
Saussureamine C	*Saussurea lappa *Clarke	58.25	49.34	207.651
5-Hydroxy-L-tryptophan	*Mucuna pruriens *	21.5	20.69	205.102
Diiodotyrosine	Ox thyroid of *Bos taurus domesticus Gmelin* (or *Bubalus bubalis L*.)	34.37	30.49	203.933
IT1t*		73.42	72.93	71.873

*Control.
